# Host traits shape flea infestation patterns in small mammals: a case study of *Spermophilus undulatus* and associated flea species in northern Xinjiang, China

**DOI:** 10.3389/fvets.2026.1783574

**Published:** 2026-03-20

**Authors:** Yiyan Zhang, Xiaoyuan Hu, Guliayi Baokaixi, Xinhui Wang, Wei Li, Qiguo Wang

**Affiliations:** 1 School of Public Health, Xinjiang Medical University, Urumqi, China; 2 Department of Emergency Response and Plague Control, Xinjiang Center for Disease Control and Prevention (Xinjiang Academy of Preventive Medicine), Urumqi, China; 3 Xinjiang Key Laboratory of Vector-borne Infectious Diseases, Urumqi, China; 4 Department of Plague Control (Labs), China Center for Disease Control and Prevention, National Institute for Communicable Disease Control and Prevention, Beijing, China

**Keywords:** host traits, hurdle negative binomial, northern Xinjiang, parasitic fleas, *Spermophilus undulatus*, *Yersinia pestis*

## Abstract

**Objective:**

Fleas are obligate ectoparasites of mammals and play an important role in the transmission of zoonotic pathogens, including *Yersinia pestis*. Describing empirical patterns of flea infestation across host communities is essential for understanding host–parasite associations in plague-endemic regions. The aim of this study is to investigate the relationship between fleas and their host animals by examining how host traits influence flea parasitism, with the goal of offering novel insights for future plague prevention strategies and related research.

**Methods:**

From 2022 to 2025, small mammals were trapped in four counties in northern Xinjiang, China. A total of 723 individuals representing eight small mammal species were examined for flea infestation, including *Spermophilus undulatus*, *Cricetulus migratorius*, *Mus musculus*, and others. Fleas were collected from hosts and the surrounding environment and identified morphologically. Host and flea diversity were quantified using Simpson’s diversity index and Sullivan’s composite diversity index. Associations between host species identity, individual-level traits, and flea infestation probability and infestation intensity were evaluated using hurdle negative binomial (HNB) regression models.

**Results:**

*Spermophilus undulatus* accounted for 65.98% of captured hosts and harbored the majority of collected fleas, primarily *Citellophilus tesquorum* and *Frontopsylla elatoides*. Flea prevalence was highest on *S. undulatus* (53.66%). HNB models indicated that flea infestation patterns were strongly associated with host species identity and selected host traits. Adult hosts exhibited higher odds of flea infestation but lower flea abundance conditional on infestation compared with immature individuals. Hosts with greater body mass carried significantly higher flea burdens, while male hosts tended to harbor more fleas than females, although this effect was marginal.

**Conclusion:**

Flea infestation patterns in northern Xinjiang are closely associated with host species identity and individual-level traits. The dominant role of *S. undulatus* in host–flea associations highlights its potential epidemiological relevance and identifies it as a key species for future pathogen surveillance within local plague foci. These findings provide an empirical description of host–flea relationships and offer ecological context for flea surveillance in plague-endemic landscapes.

## Graphical abstract

**Figure:**
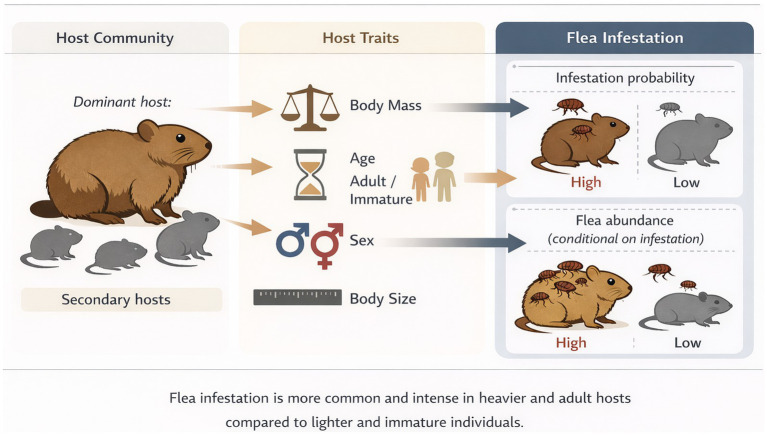

## Introduction

Fleas are obligate ectoparasites of mammals and recognized as major vectors of zoonotic pathogens, notably *Yersinia pestis*, the causative agent of plague (1–3). *Y. pestis* is primarily transmitted to humans through flea bites, with fleas infesting various small mammal species. Understanding how flea populations interact with their hosts, particularly *Spermophilus undulatus*, is crucial for assessing the risk of zoonotic transmission, especially in plague-endemic regions. While the relationship between fleas and their mammal hosts has been well documented, the potential for these fleas to harbor *Y. pestis* is a key factor in evaluating the epidemiological threat posed by rodent populations. Flea abundance and distribution are influenced by multiple ecological factors, including host diversity, density, and host-specific traits (4, 5). Variations in these factors shape flea population structure and distribution, ultimately affecting the epidemiology of flea-borne diseases.

Plague has exerted a profound influence on human history and remains enzootic in several regions worldwide, including China (6). Animal plague is reported almost annually, and sporadic human cases continue to occur (7). Human infection may result from flea bites acquired from infected small mammals or from direct contact with infected animals. Xinjiang represents one of the most important plague-endemic regions in China, with sustained epizootic activity documented in the northern Tianshan plague focus (8). The region’s heterogeneous landscapes and climatic conditions provide suitable habitats for diverse small mammal communities and their associated flea fauna, facilitating the long-term persistence of natural plague foci.

Host abundance is a fundamental determinant of parasite distribution and population size. In multi-host systems, parasite burdens are typically unevenly distributed, with a limited number of host species or individuals supporting a disproportionate share of the parasite population. Such hosts are often described as dominant or primary hosts, whereas species sustaining lower parasite burdens are regarded as auxiliary hosts. Pronounced interspecific variation in parasite abundance has been documented across diverse host–parasite systems, reflecting differences in host ecology, behavior, and habitat use (9).

However, current research is primarily focused on the ecological structure of flea communities and their interaction with environmental factors, or the direct association of immune responses (e.g., *Y. pestis* F1 antibodies) with environmental and climatic variables (10, 11). The former approach relies on cross-sectional studies of flea community structure in relation to dynamic environmental changes, which limits the ability to draw causal inferences. The latter fails to establish direct links between immune molecular factors and macroscopic environmental variables, which may contribute to ecological fallacies (12). As in the study by Mou et al. (13) although this research provides statistical associations between fleas and immune responses, it still carries a risk of ecological fallacy, especially when there is a lack of direct causal links between micro-level immunological data (F1 antibodies) and macro-level ecological data (flea index). Therefore, the study’s findings should be interpreted with caution, and more individual-level data and mechanistic research are needed to clarify the relationship between the two.

Previous studies have shown that *S. undulatus* frequently harbors high flea burdens and may play a prominent role in sustaining flea populations within plague foci, while co-occurring rodent species contribute to flea persistence to varying degrees (14, 15). The extent to which auxiliary hosts participate in flea maintenance may vary according to factors such as phylogenetic relatedness to the dominant host, habitat overlap, and individual-level characteristics. However, empirical data quantifying host–flea associations across multiple host species in northern Xinjiang remain limited.

In this study, we investigated flea infestation patterns among small mammal communities in northern Xinjiang, a region where plague remains enzootic. Using multi-year field surveillance data, we aimed to (i) describe the composition and diversity of flea assemblages across host species and sampling locations using Simpson’s Diversity Index, (ii) quantify associations between host species identity, individual-level traits, and both the probability and intensity of flea infestation using host–flea interaction networks and HNB models. By focusing on empirical patterns rather than mechanistic inference, this study seeks to provide a quantitative ecological context for understanding host-flea relationships in plague-endemic landscapes. This study does not attempt to infer causal mechanisms but focuses on describing statistically robust ecological patterns.

## Methods

### Study area

**Figure 1:**
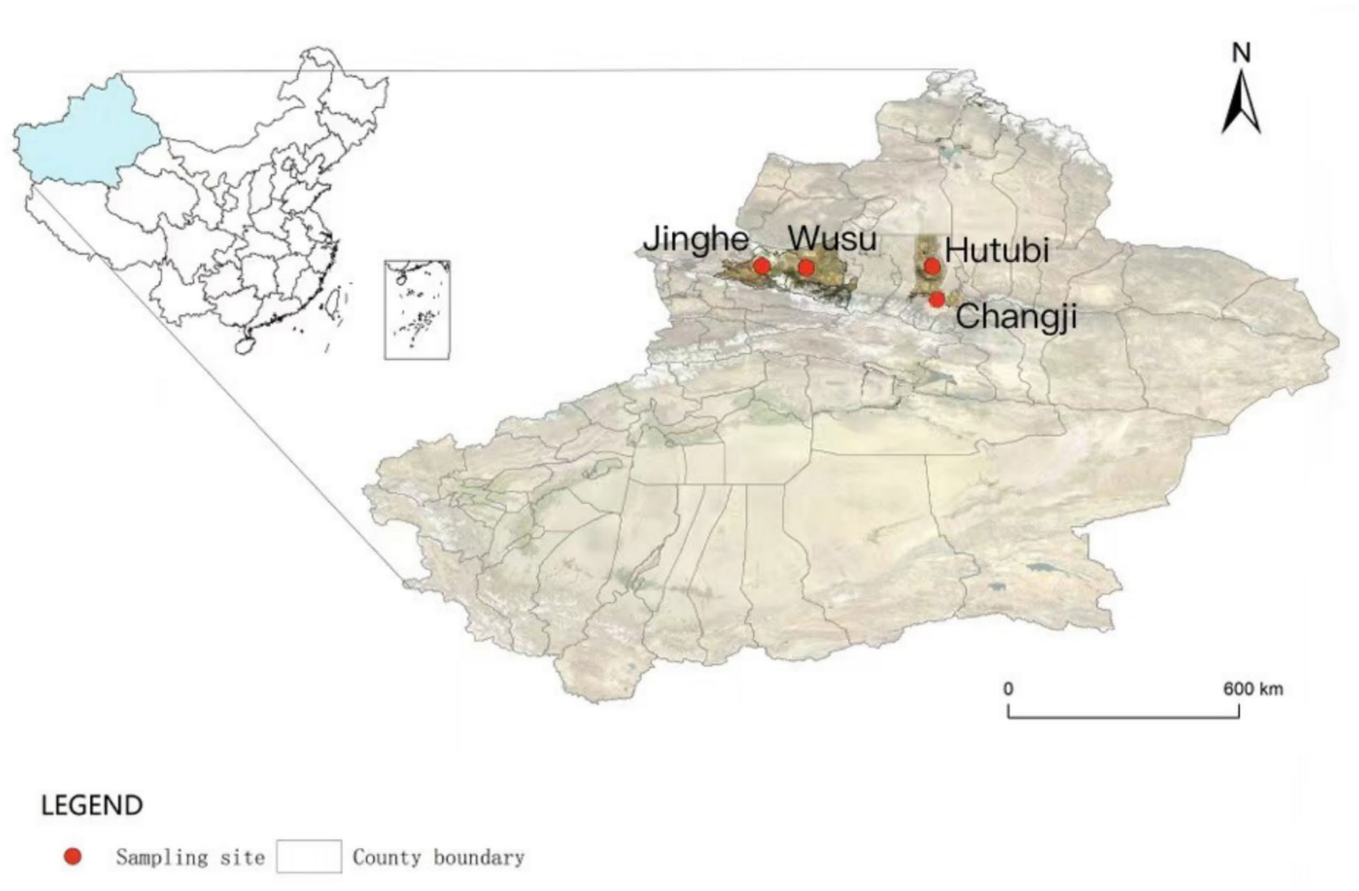
Geographic distribution of sampling counties and trapping sites included in this study. Small mammals were captured using live traps, identified to species level, and examined for flea infestation. All sampling sites are located within the study region in northern Xinjiang, China.

The study was conducted across four administrative counties in northern Xinjiang, China: Wusu, Hutubi, Changji, and Jinghe. These study units are administrative counties, not biogeographic regions. It is important to note that the contrasts between counties may reflect differences in logistics, trapping efforts, and management practices, in addition to ecological factors (16). As shown in Figure 1, small mammal trapping was conducted at four surveillance sites within the *S. undulatus* plague focus: Wusu (43°29′51″N, 82°58′42″E), Hutubi (44°11′01″N, 86°53′06″E), Changji (44°00′52″N, 87°16′03″E), Jinghe (44°36′01″N, 82°53′19″E).

### Small mammal trapping

Trapping efforts were conducted in four counties (Wusu, Hutubi, Changji, and Jinghe) across different years (2022–2025). In Jinghe and Wusu, trapping occurred annually from June to August, with additional sessions in Wusu during September in 2023. Due to logistical constraints, only one trapping session was conducted in Changji and Hutubi from June to August (2023–2025). A total of 3,500 traps were deployed across all sites. Relevant data of traps are included in the supplementary table. Small mammal trapping was conducted using Jinmao traps (Made in Guixi City, Jiangxi Province), with fried ham sausage as bait.

Fried ham sausage was used as bait. Traps were checked each morning, and traps that had captured animals were replaced with newly baited ones. Captured small mammals were euthanized by cervical dislocation and identified to species level based on morphological characteristics following established taxonomic criteria (17).

To address the unequal trapping effort across sites and years, the analysis focused on host counts only. Off-host flea data were included in descriptive figures and were not used in the regression models to avoid potential confusion.

### Flea collection and species identification

Fleas were collected from euthanized small mammals by thorough brushing of the fur and placed in securely sealed containers for transport to the laboratory, where they were processed for species identification (18). In addition, off-host fleas were collected from the environment using candle traps, which consisted of a lit candle placed at the center of a shallow plate filled with soapy water and left overnight. Fleas attracted by heat and light fell into the water and were subsequently collected and preserved in 70% ethanol (19, 20). All flea specimens were immobilized and examined under a Leica stereomicroscope. Species identification was performed based on external morphological characteristics (4, 21, 22) using published taxonomic keys (18), without clearing or slide-mounting. Both male and female specimens were identified. For flea species in which females are difficult to distinguish morphologically, particularly *Citellophilus tesquorum* and *Frontopsylla elatoides*, diagnostic characters such as spermathecal morphology were carefully examined following the procedures described by Ilinsky et al. (18). Morphological identification followed the taxonomic keys of Insecta. Siphonaptera | Fauna Sinica. Insecta. Siphonaptera (23).

### Host-flea interaction network

To evaluate whether observed host–flea associations deviated from random expectations, we performed a χ^2^ test of independence for each host–flea pair. For each test, a 2 × 2 contingency table was constructed based on the counts of the flea species across individual hosts. The four cells of the table represented: (1) number of individuals of the focal host species with the focal flea species; (2) number of individuals of the focal host species without the focal flea species; (3) number of individuals of all other host species with the focal flea species; and (4) number of individuals of all other host species without the focal flea species. When expected frequencies in any cell of the table were less than 5, we applied Fisher’s exact test instead of the χ^2^ test, or noted that results should be interpreted with caution due to small sample sizes. The edge widths are determined based on flea abundance observed in each host–flea association: line thickness is proportional to the square root of flea counts, scaled by a minimum width and a scaling factor, such that associations with higher abundance are represented by thicker edges. Specifically, the edge width is calculated as $W=0.6+6\times \sqrt{w/w_{\max}}$, where w is the number of fleas recorded for a given host–flea pair, and $w_{\max}$ is the maximum flea count across all associations. The resulting bipartite network was visualized using the R package bipartite (v. 2.19). In the network, edges are colored according to the significance level: dark orange for *p* < 0.001, orange for 0.001 < *p* < 0.05, and gray for *p* ≥ 0.05.

### Diversity indices

To quantify species diversity, Simpson’s Diversity Index was calculated following Simpson (24), which estimates the probability that two individuals randomly drawn from a community belong to different species. For each county, Simpson’s index was computed separately for host species and flea species as:


$$D=1-\sum p_{i}^{2}$$

Where $p_{i}$ represents the proportional abundance of species *i* within the community. To assess the uncertainty of these diversity estimates, 95% confidence intervals were calculated for both host and flea Simpson’s indices using a non-parametric bootstrap procedure with 10,000 resamples. To characterize multidimensional ecological diversity, we further applied Sullivan’s extension of Simpson’s index (25) as described by McLaughlin et al. (26). This composite diversity index integrates information across multiple variables—here, host species diversity and flea species diversity—and is defined as:


$$\mathrm{CDI}=1-\frac{1}{V}\sum_{k=1}^{V}\sum_{i=1}^{p}Y_{\mathrm{ki}}^{2.}$$

where V denotes the number of diversity dimensions considered (host and flea species), prepresents the number of categories within each dimension, and $Y_{\mathrm{ki}}$ is the proportional abundance of category *i* in dimension *k*. Thus, the Composite Diversity Index (CDI) quantifies the probability that two randomly selected individuals differ in at least one of the considered ecological dimensions, thereby capturing overall host–flea diversity across counties.

### Data analysis

Descriptive statistics were used to summarize the composition of small mammal communities and flea infestation patterns. Flea prevalence (presence/absence) and flea abundance (number of fleas per infested host) were calculated for each host species (27). Potential predictors of flea infestation included host species, sex, age, body mass, body length, ear length, foot length and tail length.

To account for the inclusion of multiple nocturnal rodent species with distinct morphological characteristics, continuous variables were categorized based on median values (rounded to the nearest whole number) to enhance biological interpretability. The median values were: body mass = 228 g, body length = 28 cm, ear length = 1 cm, foot length = 5 cm, tail length = 13 cm. Individuals with values ≤ the median were assigned to the reference category, and those with values > the median were assigned to the comparison category.

Associations between host-related factors and flea infestation were evaluated using hurdle negative binomial (HNB) regression models implemented in R v4.4.3 (R Foundation for Statistical Computing, Vienna, Austria) using RStudio v2022.12.0-353 (22, 28, 29). This modeling framework was selected to accommodate excess zeros and over-dispersion in flea count data. The HNB model consists of two components: (i) a logistic regression component distinguishing zero counts from positive counts (flea presence), and (ii) a zero-truncated negative binomial regression component modeling flea abundance among infested hosts.

The distribution of small mammals and flea infestations was summarized using descriptive statistics. Flea prevalence and flea abundance were calculated for each host species. Host species identity, sex, age, body mass, body length, ear length, foot length and tail length were considered as potential predictors of flea infestation patterns. Univariate HNB models were first fitted for each explanatory variable, and variables with *p* < 0.20 were retained for inclusion in the initial multivariate HNB model. A backward elimination procedure was then applied to derive the final model, using α = 0.10 as the criterion for statistical significance. Prevalence odds ratios (ORs) from the logistic component and abundance ratios (ARs) from the count component were calculated according to previously published methods (30).

## Results

### Small mammal captures

In total, 3,500 traps were deployed across four counties in northern Xinjiang, yielding 723 small mammal captures with an overall trapping success rate of 20.7%. The highest success rate was observed in Jinghe, reaching 44.8%, while Wusu had the lowest success rate of 8.6%. The captured mammals represented a variety of species, with *S. undulatus* being the most abundant.

The captured individuals represented eight species, seven genera, three families, and one order (Table 1). *S. undulatus* was the most abundant species, accounting for 65.98% (477/723) of all captures. Capture numbers differed among counties, with Jinghe yielding the highest number of individuals (*n* = 327) and Changji the lowest (*n* = 87). Species richness ranged from four to five species at the county level; Jinghe exhibited the highest richness (five species), whereas the remaining counties each contained four species.

**Table 1:** Patterns of small mammal distribution and capture rate across four counties.

County	Small mammals			Species and proportion (%) of small mammals							
	Abundance	Capture rate^a^	Richness	*S. undulatus*	*C. migratorius*	*M. musculus*	*M. libycus*	*M. meridianus*	*A. sibirica*	*D. sagitta*	*E. talpinus*
Wusu	188	5.37	4	176 (93.62)	0	0	9 (4.79)	0	0	2 (1.06)	1 (0.53)
Hutubi	121	3.49	5	89 (73.55)	0	0	18 (14.88)	12 (9.92)	0	1	1 (0.83)
Changji	87	2.49	4	67 (77.01)	8 (9.20)	4 (4.60)	0	0	8 (9.20)	0	0
Jinghe	327	9.34	4	145 (44.34)	123 (37.61)	47 (14.37)	12 (3.67)	0	0	0	0
Total	723	9.34	8	477 (65.98)	131 (18.12)	51 (7.05)	39 (5.39)	12 (1.66)	8 (1.11)	3 (0.41)	2 (0.28)

a Small mammals capture rate = (abundance/ traps) × 100%, the number of traps is 3,500. Host species were *Cricetulus migratorius* (*C. migratorius*), *Mus musculus* (*M. musculus*), *Meriones libycus* (*M. libycus*), *Meriones meridianus* (*M. meridianus*), *Allactaga sibirica* (*A. sibirica*), *Dipus sagitta* (*D.sagitta*), *Ellobius talpinus* (*E. talpinus*).

Morphological measurements were obtained from all captured individuals. Mean body mass was 227.78 g (SD = 69.24; median = 245.05 g), and mean body length was 27.54 cm (SD = 4.03; median = 28.32 cm). Additional morphometric traits, including ear length, foot length, and tail length, also showed moderate variability among individuals. These measurements were subsequently used to explore their associations with flea infestation prevalence and abundance.

### Distribution of parasitic fleas

Of the 723 small mammals examined, 296 individuals were infested with fleas, yielding an overall flea prevalence of 40.94%. A total of 1,837 fleas were collected, representing five species from five genera and two families, with a mean flea index of 2.53 fleas per host (Table 2).

**Table 2:** Prevalence and diversity of fleas among small mammal species in Xinjiang.

Host species	No. small mammals	Small mammals with flea infection	Flea prevalence^a^	Fleas	Flea index^b^	*C. tesquorum* (*n*, %)	*F. elatoides* (*n*, %)	*Neopsylla mana* (*n*, %)	*Pulex irritans* (*n*, %)	*Rhadinopsylla li* (*n*, %)
*S. undulatus*	477	256	53.66	1264	2.65	620 (49.1)	560 (44.3)	40 (3.2)	24 (1.9)	20 (1.6)
*C. migratorius*	131	2	1.53	1	0.76	1 (100)	0	0	0	0
*M. musculus*	51	20	39.22	203	3.98	20 (9.9)	150 (73.9)	15 (7.4)	12 (5.9)	6 (3.0)
*M. libycus*	39	3	7.69	4	0.1	1 (25.0)	3 (75.0)	0	0	0
*M. meridianus*	12	5	41.67	53	4.42	18 (34.0)	30 (56.6)	3 (5.7)	1 (1.9)	1 (1.9)
*A. sibirica*	8	8	100	231	28.9	0	230 (99.6)	1 (0.4)	0	0
*D. sagitta*	3	0	0	0	0	0	0	0	0	0
*E. talpinus*	2	2	100	81	40.5	2 (2.5)	78 (96.3)	0	0	1 (1.2)
Total	723	296	40.94	1837	2.53	662 (36.0)	1051 (57.2)	59 (3.2)	37 (2.0)	28 (1.5)

a Flea prevalence = (Small mammals with flea infection/Small mammals) × 100%.

b Flea index = Fleas/Small mammals.

Across all hosts, the flea assemblage was dominated by *C. tesquorum* and *F. elatoides*, which together accounted for the majority of collected specimens. Flea infestation varied markedly among host species. *S. undulatus* exhibited the highest flea prevalence (53.66%, 256/477) and harbored the largest number of fleas (*n* = 1,264), resulting in a flea index of 2.65. Both *C. tesquorum* and *F. elatoides* were frequently detected on *S. undulatus*, with relatively similar proportional representation.

In contrast, flea prevalence was low in *C. migratorius* (1.53%) and *M. libycus* (7.69%), and no fleas were detected on *D. sagitta*. Although some host species with small sample sizes, such as *A. sibirica* and *E. talpinus*, exhibited high flea indices, these estimates should be interpreted cautiously is associated with limited numbers of examined individuals.

### Host-flea interaction network

**Figure 2:**
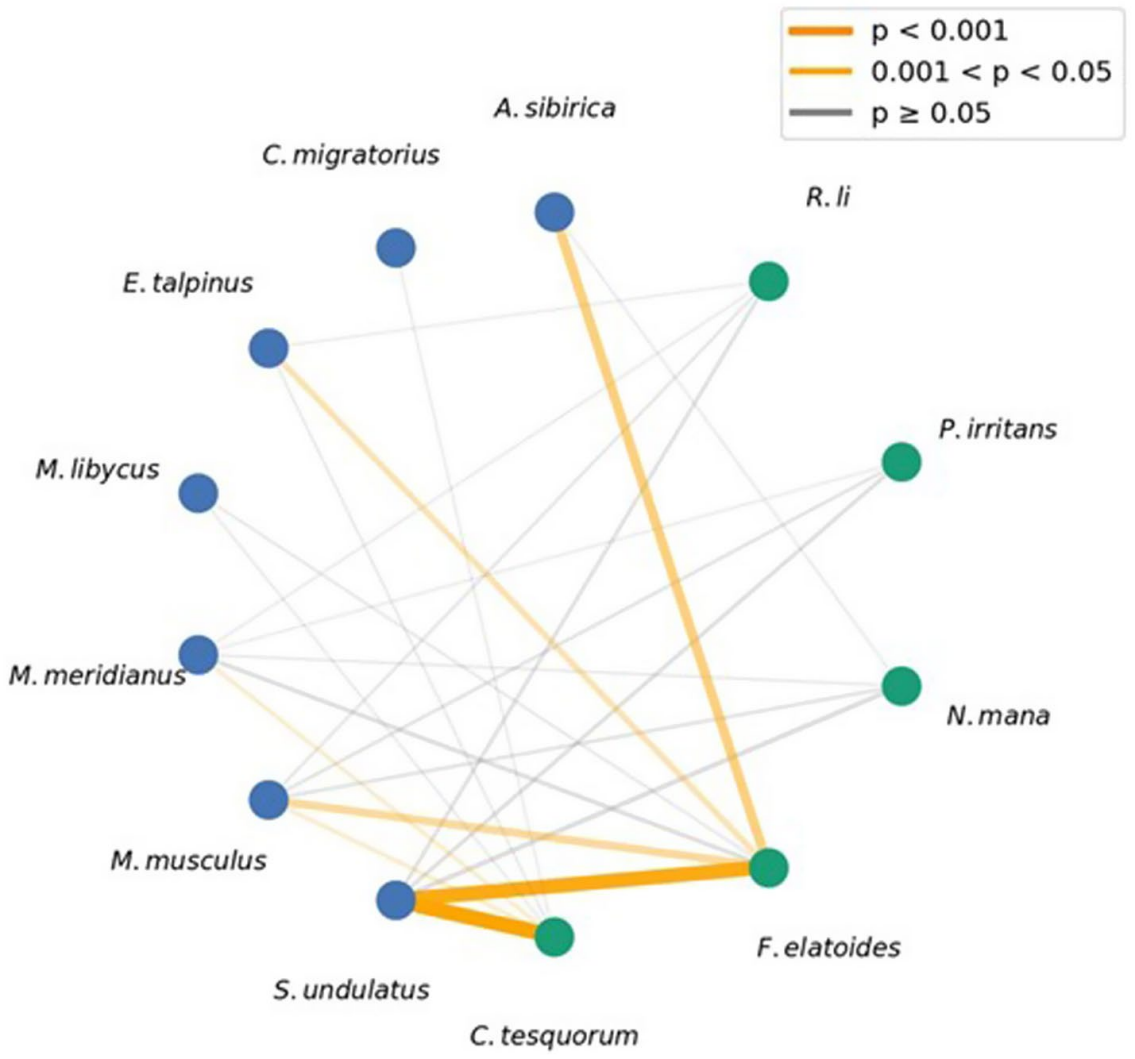
Host-flea interaction network for small mammal and flea species at the four study sites in Xinjiang. Nodes on the left represent flea species, nodes on the right represent host species. Edge width is proportional to the total number of fleas of a given species collected from a given host species. Edge color indicates the statistical significance of the association after FDR correction, based on a 2 × 2 contingency table test of presence/absence across host individuals.

The host–flea interaction network (Figure 2) exhibited a heterogeneous structure, with interactions unevenly distributed among host species. A global χ^2^ test confirmed a significant overall association between host and flea communities (χ^2^ = 398.04, df = 28, *p* < 0.001), indicating that flea species are non-randomly distributed across host species. *S. undulatus* showed the highest connectivity, being associated with all five recorded flea species and accounting for the majority of observed host–flea interactions. Notably, highly significant associations (FDR adjusted *p* < 0.001) were observed between *S. undulatus* and the dominant flea species, *C. tesquorum* and *F. elatoides*, with edge widths reflecting the high flea counts (620 and 560 individuals, respectively).

In contrast, other host species displayed fewer interactions and lower levels of connectivity within the network. *M. musculus* was linked to multiple flea species but contributed relatively few interactions overall, with significant associations (*p* < 0.05) for *C. tesquorum* and *F. elatoides*. *A. sibirica* showed a highly significant association with *F. elatoides* (*p* < 0.001), despite its small sample size, as reflected by the wide edge in the network. *D. sagitta* showed no detectable host–flea associations and was excluded from the network.

### Diversity patterns across counties

**Figure 3:**
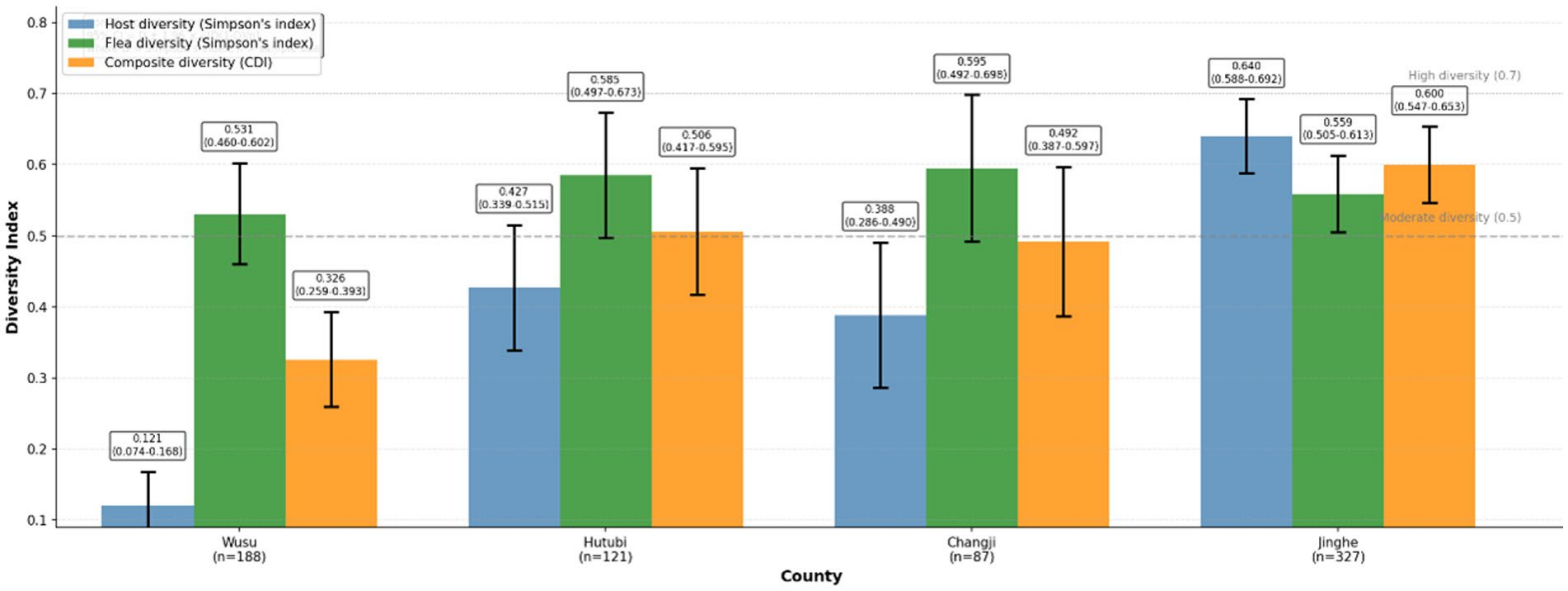
Diversity patterns of small mammal hosts and parasitic fleas across sampling counties. Simpson diversity indices were calculated separately for hosts and fleas, and a composite diversity index (CDI) was used to characterize overall host–parasite community diversity. Points represent point estimates; error bars indicate 95% confidence intervals calculated using non-parametric bootstrap (10,000 resamples). Higher values indicate greater diversity.

The composite diversity index (CDI) was calculated using Sullivan’s extension of Simpson’s index, accounting for both host and flea diversity across counties. Flea infestation was highest in *S. undulatus*, though other host species, such as *C. migratorius* and *M. libycus*, also contributed to the overall flea burden. Simpson’s diversity index for small mammal hosts ranged from 0.121 (95% CI: 0.079–0.163) in Wusu to 0.640 (95% CI: 0.588–0.692) in Jinghe. Flea diversity showed a different pattern, with Simpson’s indices ranging from 0.531 (95% CI: 0.460–0.602) in Wusu to 0.595 (95% CI: 0.492–0.698) in Changji, demonstrating relatively stable flea assemblages across the study region despite marked variation in host communities (Figure 3).

When host and flea diversity were combined using the composite diversity index (CDI), Jinghe exhibited the highest overall diversity (CDI = 0.600, 95% CI: 0.547–0.653), followed by Wusu (CDI = 0.506, 95% CI: 0.417–0.595) and Hutubi (CDI = 0.506, 95% CI: 0.417–0.595), while Changji showed the lowest composite diversity (CDI = 0.492, 95% CI: 0.387–0.597). These results reveal spatial variation in host community composition coupled with relatively conserved flea assemblages, highlighting the dominant role of *S. undulatus* in shaping local host–parasite community structure.

### Determinants of flea infestation and abundance

Host species was the strongest predictor of flea infestation patterns. *S. undulatus* had a much higher probability of flea infestation compared to *C. migratorius* (*p* < 0.001). Other host species showed lower infestation odds but significantly higher flea abundance when infested by fleas.

Individual traits also influenced flea infestation. Adults were more likely to be infested than immature individuals, though the effect was not statistically significant. However, adults harbored fewer fleas than juveniles. Heavier hosts (>228 g) carried significantly more fleas (*p* < 0.001), while body mass did not influence infestation odds. Sex had some effect on flea abundance, with male hosts generally carrying more fleas, but this difference was not statistically significant.

**Figure 4:**
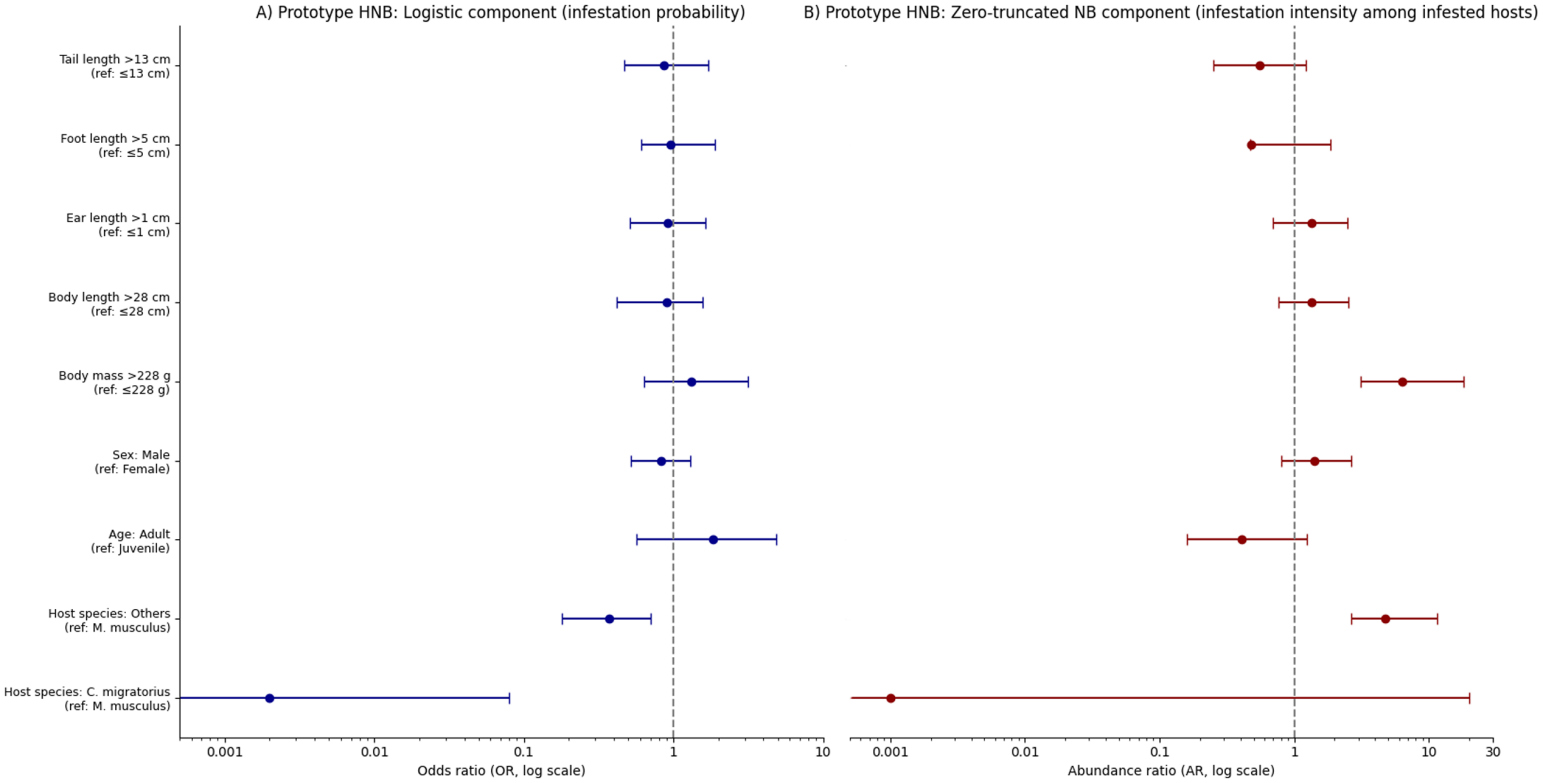
Prototype hurdle negative binomial (HNB) regression analysis of flea infestation patterns. **(A)** Logistic component (infestation probability); **(B)** Zero-truncated negative binomial component (infestation intensity among infested hosts).

**Figure 5:**
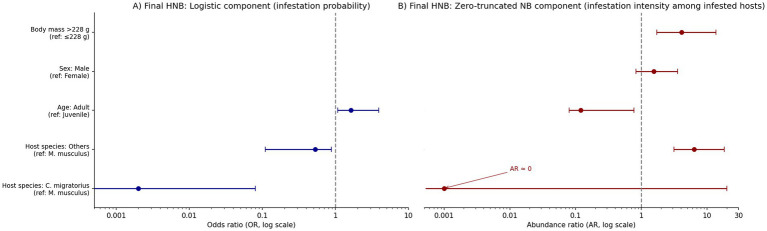
Final parsimonious hurdle negative binomial (HNB) regression analysis of flea infestation patterns. **(A)** Logistic component (infestation probability); **(B)** Zero-truncated negative binomial component (infestation intensity among infested hosts).

The final model retained host species, age, sex, and body mass as significant predictors. Adults exhibited higher odds of flea infestation but lower flea abundance compared to juveniles, and heavier hosts carried more fleas (Figures 4, 5; Tables 3, 4).

**Table 3:** Output of the prototype multivariate negative binomial model modeling parasitic flea abundance patterns.

Variables (factors)		Small mammals	Logistic component (infestation probability)		Zero-truncated negative binomial component (infestation intensity among infested hosts)	
			OR (95%CI)	*P* value	AR (95%CI)	*P* value
Host species	*S. undulatus*	477	Ref^a^		Ref	
	*C. migratorius*	131	0.002 (0, 0.08)	<0.001	0 (0, + ∞)	0.963
	Others	115	0.37 (0.18, 0.71)	<0.001	4.75 (2.66, 11.45)	<0.001
Age	Immaturity	203	Ref		Ref	
	Adult	520	1.83 (0.57, 4.88)	0.138	0.41 (0.16, 1.24)	0.069
Sex	Female	453	Ref		Ref	
	Male	270	0.83 (0.52, 1.29)	0.371	1.42 (0.81, 2.67)	0.192
Body mass (g)	≤228	359	Ref		Ref	
	>228	364	1.31 (0.64, 3.14)	0.577	6.32 (3.14, 18.23)	<0.001
Body length (cm)	≤28	364	Ref		Ref	
	>28	359	0.90 (0.42, 1.57)	0.583	1.34 (0.76, 2.53)	0.321
Ear length (cm)	≤1	382	Ref		Ref	
	>1	341	0.92 (0.51, 1.64)	0.862	0.51 (0.69, 2.48)	0.221
Foot length (cm)	≤5	396	Ref		Ref	
	>5	327	0.96 (0.61, 1.89)	0.847	0.48 (0.47, 1.85)	0.199
Tail length (cm)	≤13	357	Ref		Ref	
	>13	366	0.86 (0.47, 1.72)	0.673	0.55 (0.25, 1.22)	0.154

a Ref: reference group.

**Table 4:** Output of the finalized multivariate HNB model modeling parasitic flea abundance patterns.

Variables (factors)		Logistic component (infestation probability)		Zero-truncated negative binomial component (infestation intensity among infested hosts)	
		OR (95%CI)	*P* value	AR (95%CI)	*P* value
Host species	*S. undulatus*	Ref^a^		Ref	
	*C. migratorius*	0.002 (0, 0.08)	<0.001	0 (0, + ∞)	0.813
	Others	0.53 (0.11, 0.88)	<0.001	6.32 (3.14, 18.23)	<0.001
Age	Immaturity	Ref		Ref	
	Adult	1.63 (1.07, 3.92)	0.031	0.12 (0.08, 0.77)	<0.001
Sex	Female			Ref	
	Male			1.54 (0.83, 3.57)	0.072
Body mass (g)	≤228			Ref	
	>228			4.11 (1.71, 13.59)	< 0.001

a Ref: reference group.

## Discussion

This study provides novel insights into the flea vector ecology within a key plague focus of northern Xinjiang, with epidemiological implications centered on the Siberian suslik (*S. undulatus*) as a principal host in the local host-flea network. *C. tesquorum* and *F. elatoides*, whose preferential association with *S. undulatus* underscores this rodent’s pivotal role in sustaining the local vector population. Beyond refining the known geographic distribution of *S. undulatus*, our findings elucidate its function as a hub species within the host–parasite network, facilitating the co-occurrence of multiple flea taxa. This pattern of host-sharing among vectors, particularly involving known plague vectors, may have critical implications for interspecific transmission dynamics and the persistence of *Yersinia pestis* within the ecosystem. Given that flea infestations are contingent on host availability, a clear understanding of small mammal diversity and abundance is fundamental for evaluating potential vector-host dynamics. The Simpson’s diversity indices for fleas across our four study counties showed relative stability, ranging from 0.531 to 0.595, consistent with prior surveillance in northern Xinjiang that also reported stable flea diversity across sampling sites (31). This stability stands in contrast to the marked spatial heterogeneity we observed in host diversity (Host Simpson’s index range: 0.121–0.640). The broader confidence intervals for host diversity estimates (e.g., Wusu: 0.074–0.168; Jinghe: 0.588–0.692) suggest greater uncertainty in host community assessments compared to flea assemblages, likely reflecting differences in host community structure driven by habitat heterogeneity and resource availability across counties.

Previous work has emphasized how flea species richness and composition influence the persistence of *Y. pestis* (32); therefore, despite the moderate and spatially consistent flea diversity reported here, targeted pathogen screening of dominant flea species remains crucial for epidemiological risk assessment. Research on *S. undulatus* and its parasites in northwestern China has identified fleas as carriers of *Bartonella* and other pathogens (14); As *S. undulatus* constituted a major portion of our host samples (65.98% of all captures), the low host diversity at some sites (e.g., Wusu: Simpson’s index = 0.121) suggests potential dominance by a few competent reservoir species, a scenario that could intensify local transmission dynamics through reduced host community dilution effects.

Our findings confirm *S. undulatus* as the predominant rodent species in this region and the principal host for the dominant flea species, *C. tesquorum* and *F. elatoides*. This aligns with earlier reports identifying *S. undulatus* as the primary rodent in commensal plague foci, with *C. tesquorum* and *F. elatoides* as the key parasitic fleas (31). Within these foci, *S. undulatus* serves as the main host, while the steppe lemming, *C. migratorius*, functions as an auxiliary host. As posited by Hammond et al. (33), flea infestation intensity is closely tied to host species. Our data further reveal that *C. tesquorum* infestation rates were higher on *S. undulatus* than on *C. migratorius* or other hosts, indicating that *S. undulatus* is the most suitable species for flea proliferation. Consequently, the probability of heavy flea infestation on species like *M. musculus* and other rodents appears comparatively lower.

The increased flea infestation intensity observed on auxiliary hosts like *C. migratorius*, despite their lower population densities, may be attributable to high parasite loads sustained by individual hosts. Disparities in parasitic infestation intensity between primary and auxiliary hosts often stem from differences in parasite exploitation efficiency and reproductive success (34). The phylogenetic relatedness between primary and auxiliary hosts can further influence parasite infestation intensity on the latter, reflecting shared physiological, ecological, and immunological traits (35). Moreover, flea infestation levels on auxiliary hosts typically decrease as phylogenetic distance from the main host increases (36).

Our analysis of both the prototype and final hurdle negative-binomial (HNB) models indicates that host identity, body mass, and demographic state are primary determinants of flea infestation probability and intensity within the Tianshan small-mammal assemblage. Notably, *C. migratorius* exhibited a dramatically lower probability of infestation relative to *S. undulatus*, whereas “other” host species, despite lower infestation odds, harbored substantially higher flea infestation intensity when infested (final model: OR ≈ 0.002 for *C. migratorius*; OR ≈ 0.53 and AR ≈ 6.32 for other species). These patterns reflect the well-documented taxa-specific effects on parasite prevalence and intensity, in which host species identity plays a central role.

Differences in behavior, microhabitat use, and immune defenses account for a considerable portion of the observed variation in parasite burdens. Recent studies have further shown that parasites preferentially exploit hosts that maximize their reproductive success (37). Consequently, host selection emerges as a critical factor influencing both flea infestation intensity and assessments of potential flea-mediated disease risk. Moreover, infection levels often vary among individual hosts, with certain individuals representing more favorable “patches” for parasites than others (38). By definition, hosts providing the most suitable microhabitats for parasite survival and reproduction are considered primary hosts.

The positive association between host body mass and flea load observed in our study, where hosts weighing over 228 g carried approximately five times more fleas is consistent with both theoretical predictions and empirical observations. Larger hosts offer increased habitat and resource availability for parasites, often supporting greater parasite biomass or richness. Such scaling relationships between host size and parasite load have been documented across diverse host–parasite systems. Accordingly, host body mass can serve as a practical proxy for potential flea assemblages. In our study, flea infestation intensity increased in small mammals exceeding 228 g. Similarly, Okabe et al. (39) reported that flea numbers, particularly *C. tesquorum* and *F. elatoides*, in wild rodents rose with host mass (and presumably age), reaching an optimum before declining. Since host body mass generally increases with age, and the probability of flea infestation also rises with host age, these factors collectively contribute to the observed positive correlation between host mass and flea infestation intensity.

*Citellophilus tesquorum* was found to infest *S. undulatus* predominantly, while also parasitizing other small mammal species, indicating limited host specificity. Such a broad host range has likewise been documented across the Central Asian continent (18), suggesting a consistent ecological pattern at a broader geographic scale. The frequent co-occurrence of *C. tesquorum* with *F. elatoides* raises particular concern regarding its potential role in plague transmission. Accordingly, further investigation is required to evaluate its capacity to harbor *Y. pestis* and to assess its vector competence relative to established plague vectors.

We observed opposing age effects on flea infestation: adults were more likely to be infested (OR > 1), yet, conditional on infestation, they harbored fewer fleas (AR < 1) than juveniles. This pattern aligns with previous findings suggesting that adults may experience higher encounter rates or susceptibility due to behavior or exposure, whereas juveniles, potentially owing to immature immunity or parental aggregation, sustain higher parasite intensities per infested individual. Such stage-specific dynamics between infestation probability and infestation intensity have been highlighted in field studies of parasites and in reviews of host ontogeny and parasitism.

Age-related differences in flea infestation intensity may be influenced by variations in parasite aggregation. Our analyses indicate that, while the probability of flea infestation increases with host age, the actual number of fleas decreases in adults. This phenomenon is generally linked to host immune function and parasite–host interactions. Immune defenses often deteriorate with age (40), and in small mammals, antiparasitic defenses have been reported to decline with increasing age (41), affecting the volume of blood a flea can acquire (42). Fleas have been observed to ingest more blood from juveniles and older individuals compared to sub-adults and adults (43). The rate of immune senescence, however, may be modulated by environmental conditions such as stress and may differ between sexes due to faster aging in males (44).

Differential parasite infestation intensity across host age cohorts has been documented in various host–parasite systems (45), although the influence of age on parasite distribution varies. In some associations, infestation intensity increases with host age (46), whereas in others, it is elevated in the youngest and oldest hosts relative to median-age individuals (45). Consequently, these age groups provide more favorable “habitats” for parasites, producing a convex relationship between infestation intensity and host age. If heavy parasite loads preferentially increase mortality in juveniles rather than older hosts, infestation intensity may rise with host age. Overall, fleas tend to perform better in the youngest and oldest hosts compared to middle-aged individuals.

Regarding sex-specific differences, our results indicate a tendency toward higher flea infestation intensity in male hosts, although this effect was not statistically significant in the final model. We examined the influence of host sex on flea infestation and observed that small male mammals harbored more fleas than females. Understanding the impact of host sex is critical for elucidating the mechanisms underlying male-biased parasitism. This observed male-biased pattern is consistent with broad comparative evidence showing that males often carry higher parasite loads, a phenomenon commonly attributed to sex-related differences in hormone levels, immune function, and behavior, although the magnitude and significance of this effect vary across systems (47).

Under normal conditions, two non-mutually exclusive mechanisms have been proposed to explain variation in flea infestation intensity. Male hosts typically exhibit higher mobility and comparatively weaker immune defenses than females. Fleas feeding on males generally ingest blood more rapidly, consume larger volumes, and digest it more quickly, although the pattern of blood digestion can be influenced by external factors such as relative humidity. Additionally, fleas produce more eggs when exploiting male hosts than females (47). Collectively, these factors enhance the reproductive success of fleas on male hosts, likely driven by the immunosuppressive effects of androgens, which may lead fleas to preferentially exploit males.

Associations between flea aggregation and host traits are shaped by multiple interacting factors. Our study confirms that variation in flea numbers is largely attributable to host-related traits, underscoring the importance of host identity when comparing flea dynamics in natural rodent populations. These findings highlight the need to incorporate host factors into strategies aimed at regulating flea infestation intensity. In practice, fitness-related measures, such as reducing host densities and limiting flea populations, are essential for the prevention and control of flea-borne diseases.

## Limitations

This study has several limitations. First, flea identification was based solely on morphology; molecular approaches (e.g., DNA barcoding) would improve species resolution for challenging taxa such as *C. tesquorum* and *F. elatoides*. Second, no positive detection of *Y. pestis* F1 antibodies in hosts was obtained, limiting direct epidemiological inference; detecting positive samples would require approximately 5,000–6,000 host animals, suggesting larger sample sizes for future studies. Third, the observed associations are correlational; experimental studies are needed to establish causality between host traits and flea burdens. Fourth, dichotomizing continuous variables may have oversimplified underlying relationships. Fifth, our modeling approach has two important methodological limitations. First, the models did not account for potential clustering of individuals within counties or sampling sessions, which may have produced overly narrow confidence intervals and optimistic *p*-values; future studies with hierarchical designs should employ mixed-effects models to address this non-independence. Second, the use of univariate screening (*p* < 0.20) followed by backward stepwise selection may introduce model selection bias and yield overly optimistic inferences. Given the exploratory nature of this study, these model-building choices should be considered hypothesis-generating, and the final selected model requires validation in independent datasets.

## Future directions

Building on the findings and limitations outlined above, several key avenues for future research are recommended to advance the understanding of flea-borne disease ecology in northern Xinjiang and similar plague foci:

(1) Integration of molecular taxonomy and pathogen surveillance

Future studies should integrate morphological and molecular methods for flea identification. DNA barcoding can validate challenging taxa (e.g., *C. tesquorum*, *F. elatoides*) and detect cryptic species. Concurrent pathogen screening for *Y. pestis*, *Bartonella* spp., and *Rickettsia* spp. in dominant flea vectors is essential to assess their vector competence and microbial burden.

(2) Mechanistic and experimental studies

To move beyond correlational patterns, controlled experiments and field studies are needed to investigate how host age, sex, and body condition affect flea feeding and reproduction and conduct host-choice experiments to understand flea preferences. Expanding host sample size to 5,000–6,000 individuals will ensure statistical power.

(3) Longitudinal and landscape-level monitoring

Establishing long-term monitoring sites across environmental gradients will enable tracking of seasonal variation in flea infestation intensity and inter-annual host fluctuations. Integrating landscape metrics can reveal how environmental drivers modulate disease risk.

(4) Advanced analytical frameworks

Hierarchical models (e.g., mixed-effects models) should address nested data structures, while generalized additive models (GAMs) can explore non-linear relationships between host traits and flea loads. Larger datasets (5,000–6,000 hosts) will improve model robustness and predictive accuracy for flea-borne disease dynamics.

## Conclusion

In summary, our study demonstrates that flea infestation patterns within the northern Xinjiang plague focus are strongly determined by host species identity and key individual traits, notably body mass and age. *S. undulatus* emerged unequivocally as the dominant host for the primary flea vectors, *C. tesquorum* and *F. elatoides*, while co-occurring rodent species played auxiliary roles in the flea community. These findings extend beyond a basic ecological description; they identify a high-risk host–parasite complex central to the local flea community, whose role in pathogen transmission warrants further investigation. Specifically, they highlight that monitoring programs aimed at understanding flea-borne disease risk should prioritize

*Spermophilus undulatus* populations and quantify flea burdens, with particular attention to larger individuals which may disproportionately contribute to vector maintenance.

The integration of host trait data into ectoparasite distribution models, as validated here, enhances the precision of ecological characterization of host–parasite associations. Ultimately, translating these strong ecological associations into actionable epidemiological intelligence requires a critical next step: direct pathogen screening of the dominant flea vectors for *Y. pestis* and other zoonotic agents. Future research combining such molecular surveillance with mechanistic studies on vector competence and host immunology will be essential to fully evaluate the transmission potential inherent in these documented host-flea relationships.

## Data Availability

The original contributions presented in the study are included in the article/supplementary material, further inquiries can be directed to the corresponding author.
