# Supplementary figures and images for: Host traits shape flea infestation patterns in small mammals: a case study of Spermophilus undulatus and associated flea species in northern Xinjiang, China
Source: Front Vet Sci. 2026 Mar 20;13:1783574. doi: 10.3389/fvets.2026.1783574 (PMC13047838; doi:10.3389/fvets.2026.1783574)

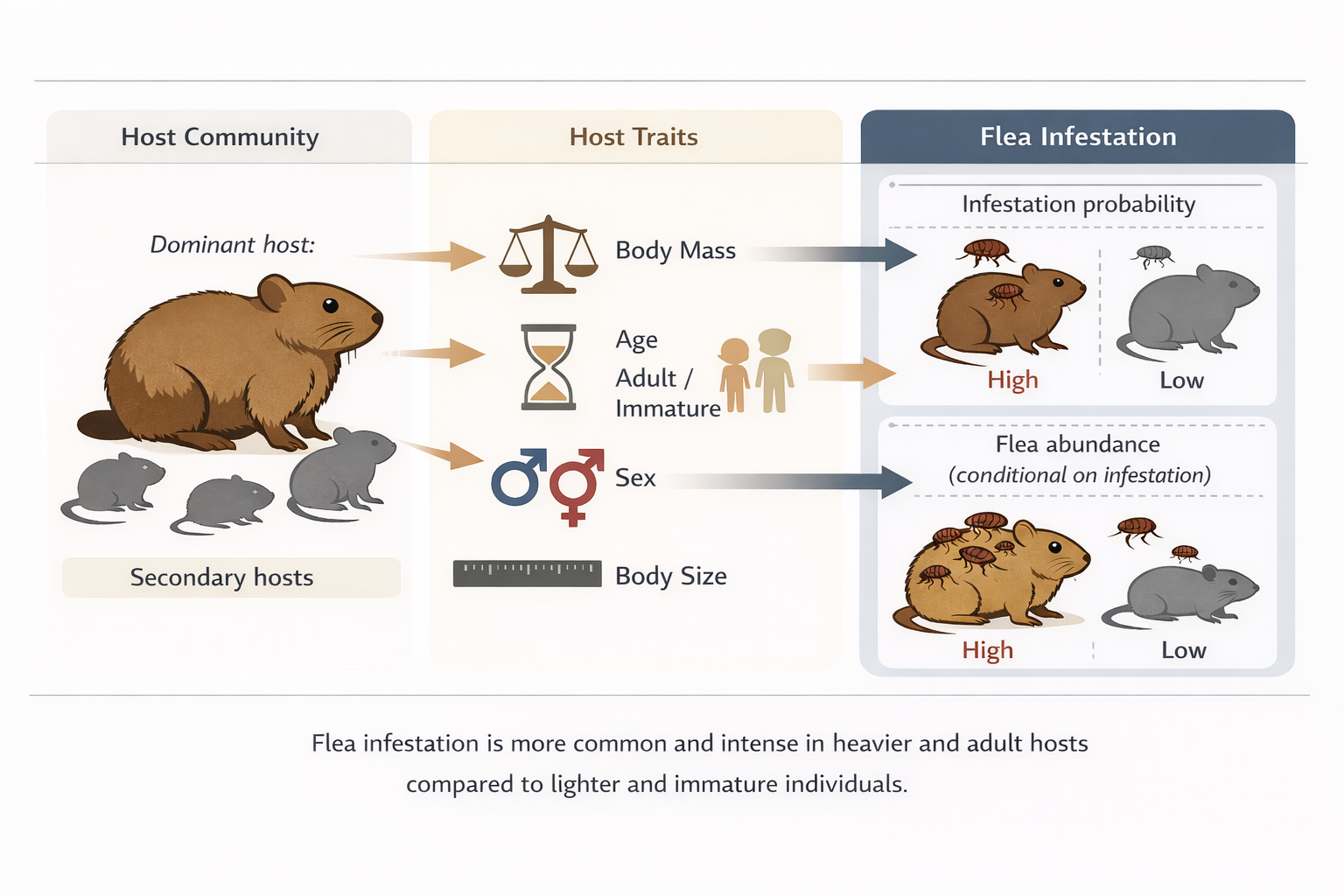

Supplement: Supplementary file 1 [file Image_1.PNG]

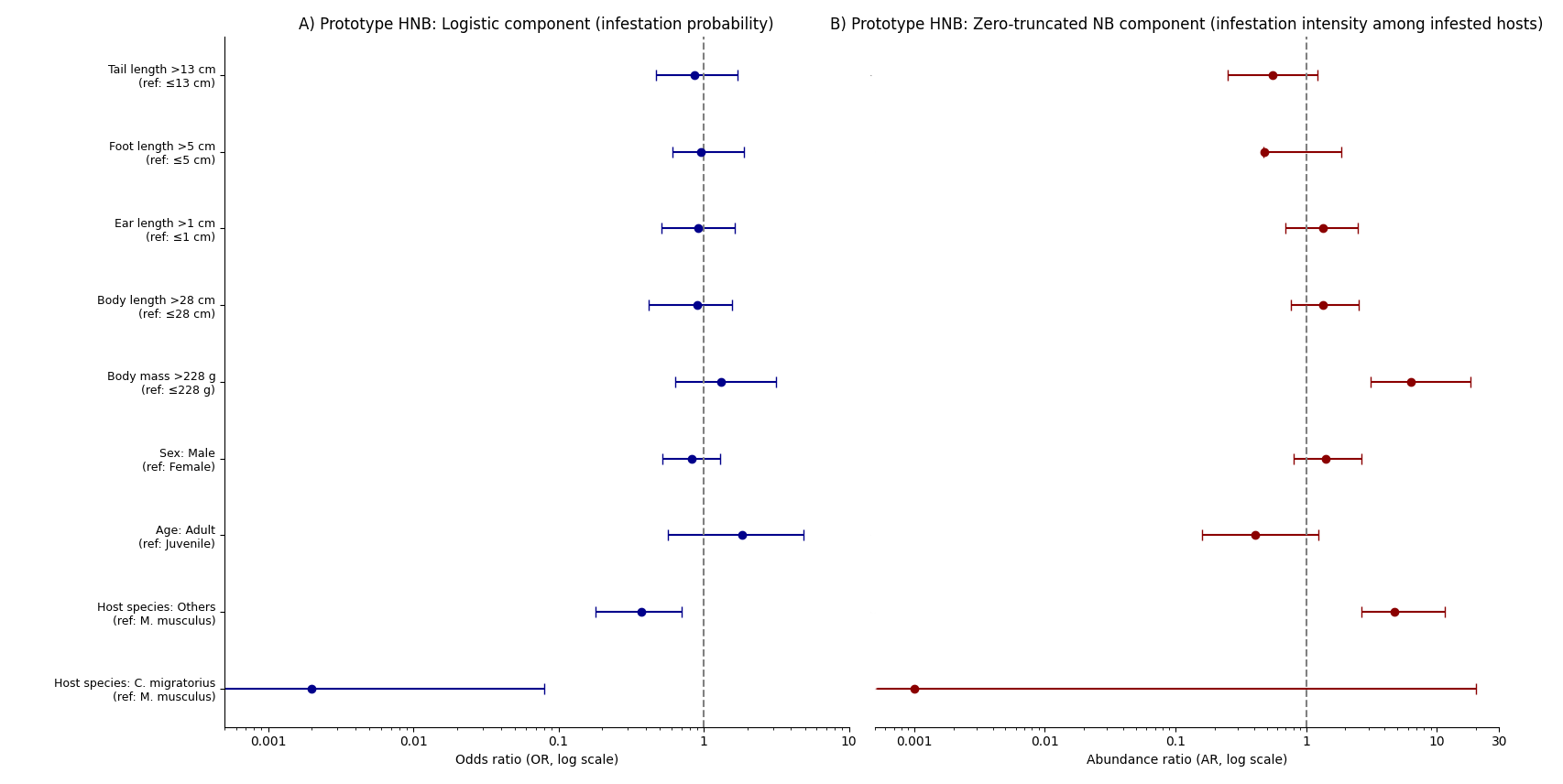

Supplement: Supplementary file 2 [file Image_2.PNG]

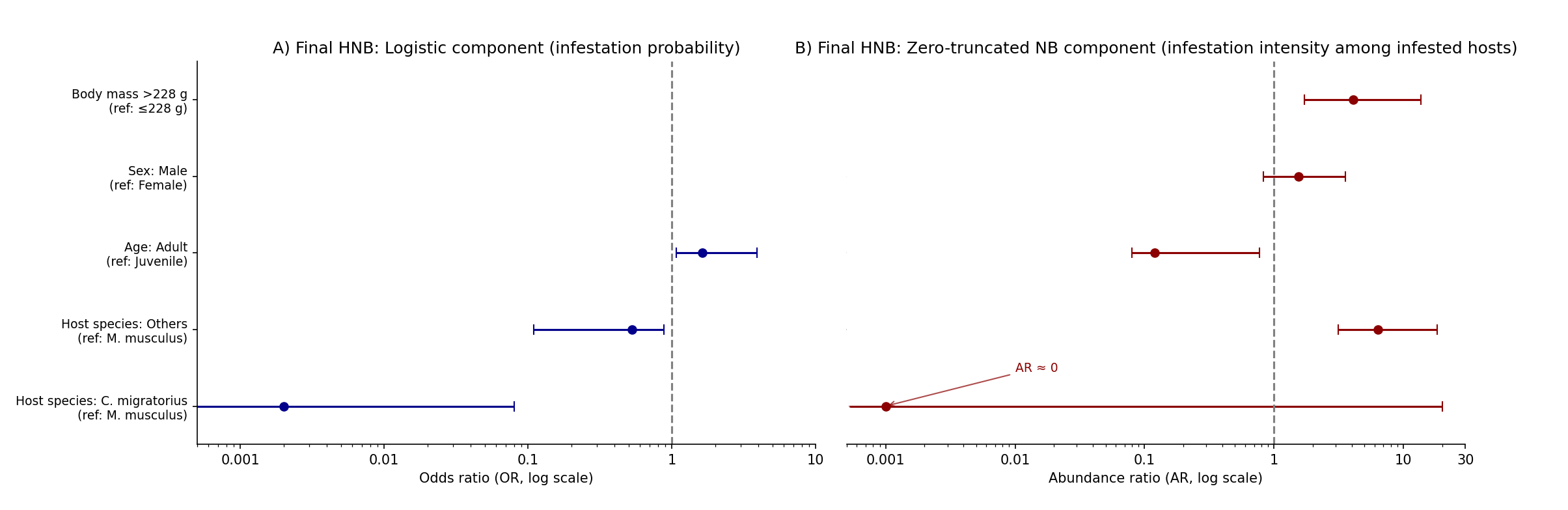

Supplement: Supplementary file 3 [file Image_3.PNG]

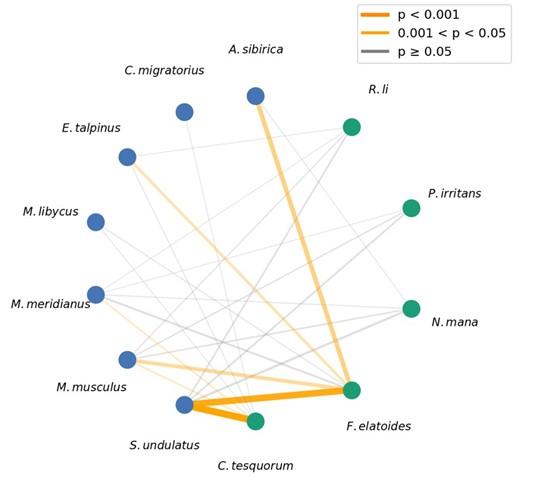

Supplement: Supplementary file 4 [file Image_4.JPEG]

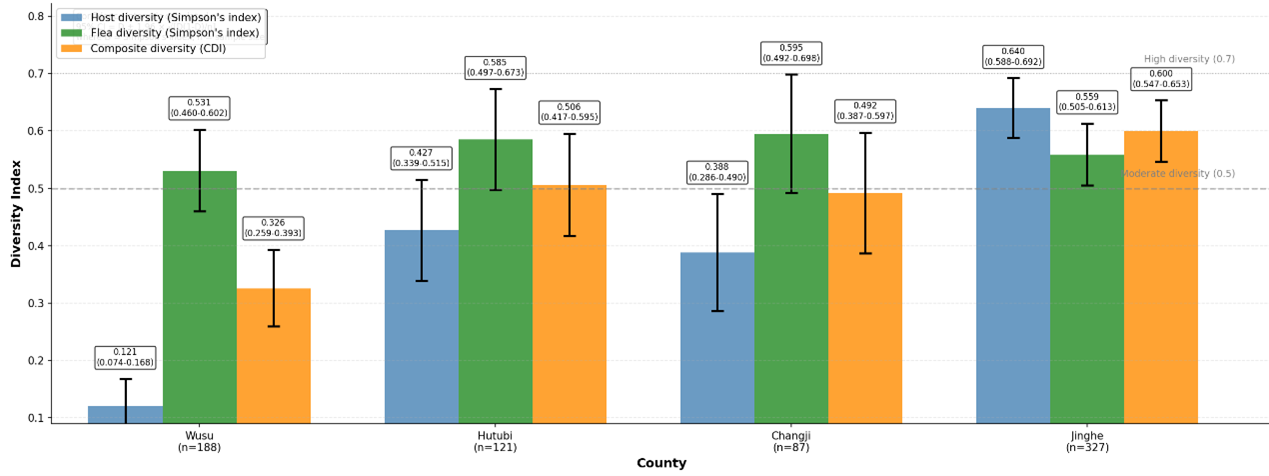

Supplement: Supplementary file 5 [file Image_5.PNG]
