# Supplementary material for: Host traits shape flea infestation patterns in small mammals: a case study of Spermophilus undulatus and associated flea species in northern Xinjiang, China
Source: Front Vet Sci. 2026 Mar 20;13:1783574. doi: 10.3389/fvets.2026.1783574 (PMC13047838; doi:10.3389/fvets.2026.1783574)
Supplement: Supplementary file 6 [file Table_1.DOCX]

**Supplementary Materials for the main article**

A total of 3,500 traps were deployed across four counties in northern Xinjiang, resulting in the capture of 723 small mammals and an overall trapping success of 20.66% (Table 1)

**Table 1.**Sampling information and small mammal capture results for Wusu, Hutubi,

Changji, and Jinghe.

| Country | Sampling periods | Nights of capture | Traps | ^a^Trapping efforts | Trapped mammals | bTrapping success (%) |  |
| --- | --- | --- | --- | --- | --- | --- | --- |
|  |  |  |  |  |  |  |  |
|  |  |  |  |  |  |  |  |
| Wusu | Jun. 2022 | 5 | 100 | 500 | 43 | 8.6 |  |
|  | Jun. 2023 | 5 | 100 | 500 | 57 | 11.4 |  |
|  | Jul. 2025 | 5 | 100 | 500 | 88 | 17.6 |  |
| Total Wusu |  | 15 | 300 | 1500 | 188 | 37.6 |  |
| Hutubi | Jun. 2023 | 5 | 100 | 500 | 121 | 24.2 |  |
| Total Hutubi |  | 5 | 100 | 500 | 121 | 24.2 |  |
| Changji | Jul. 2024 | 5 | 100 | 500 | 87 | 17.4 |  |
| Total Changji |  | 5 | 100 | 500 | 87 | 17.4 |  |
| Jinghe | Jun. 2024 | 5 | 100 | 500 | 103 | 20.6 |  |
|  | Jul.-Aug. 2025 | 5 | 100 | 500 | 224 | 44.8 |  |
| Total Jinghe |  | 10 | 200 | 1000 | 327 | 32.7 |  |
| Overall total |  | 35 | 700 | 3500 | 723 | 20.7 |  |

^a^Trapping efforts = Nights of capture × Traps, ^b^Trapping success = (Trapped mammals/ Trapping efforts) × 100%
